# Artificial intelligence and big data for precision regenerative medicine in knee osteoarthritis: endotyping, responder prediction, and clinical translation

**DOI:** 10.3389/fbioe.2026.1899487

**Published:** 2026-07-15

**Authors:** Lichuan Zheng, Jiayi Li, Hai Wang, Jiahui Zhou, Pengfei Zhao, Qi Li, Yikui Zhang, Yong Zheng

**Affiliations:** 1 Department of Orthopedics, BingTuanSiShi Hospital, Yining City, China; 2 School of Medicine, Nankai University, Tianjin, China

**Keywords:** clinical decision support systems, knee osteoarthritis, machine learning, multimodal data, orthobiologics, platelet-rich plasma, precision medicine

## Abstract

Knee osteoarthritis (KOA) is a heterogeneous whole-joint disease, and regenerative and orthobiologic therapies such as platelet-rich plasma (PRP), mesenchymal stem cells (MSCs), bone marrow aspirate concentrate (BMAC), microfragmented adipose tissue (MFAT), and extracellular vesicles (EVs) show variable clinical effects. This variability reflects a dual heterogeneity: patients differ in structural damage, inflammation, metabolism, biomechanics, pain mechanisms, and molecular endotypes, while therapeutic products differ in composition, dose, viability, secretome, and manufacturing protocols. This Mini Review discusses how multimodal characterization of both patients and products may provide the data foundation for precision regenerative medicine in KOA. Imaging, radiomics, biomechanics, multi-omics, and product-quality attributes can be integrated to define meaningful endotypes and support responder prediction. We critically evaluate current artificial intelligence (AI) applications and demonstrate that, although AI has advanced automated imaging assessment and KOA progression prediction, direct evidence for regenerative treatment-response prediction remains scarce. Existing models are largely limited to PRP, whereas validated AI models for MSC-, BMAC-, MFAT-, and EV-based therapies are lacking. Clinical translation will require more than high discrimination metrics. Explainable AI, calibration, uncertainty estimation, external and prospective validation, standardized product reporting, and clinical decision support integration are essential. Future progress depends on matched patient–product–outcome cohorts that enable adaptive, explainable, and clinically actionable treatment selection.

## 1 Introduction

Knee osteoarthritis (KOA) is increasingly recognized as a heterogeneous whole-joint disease involving structural, inflammatory, metabolic, biomechanical, and pain-related mechanisms (Tang et al., 2025). Although total knee arthroplasty remains effective for end-stage disease, there is a substantial unmet need for earlier interventions capable of modifying the intra-articular environment and potentially slowing structural deterioration. Consequently, orthobiologic and cell-based therapies, including platelet-rich plasma (PRP), mesenchymal stem cells (MSCs), bone marrow aspirate concentrate (BMAC), microfragmented adipose tissue (MFAT), and extracellular vesicles (EVs), have attracted considerable interest. Rather than merely suppressing pain, these interventions aim to modulate inflammation, promote joint homeostasis, and support tissue repair (Andia et al., 2025; Goulian et al., 2025).

Despite a strong biological rationale, the clinical translation of regenerative therapies for KOA is limited by highly inconsistent outcomes across trials and patient populations. For example, the RESTORE randomized clinical trial showed that intra-articular PRP was not superior to saline placebo for 12-month symptom improvement or medial tibial cartilage volume preservation in mild-to-moderate KOA (Bennell et al., 2021). This result highlights the gap between biological plausibility and reproducible clinical efficacy, particularly when heterogeneous patients are treated under variable protocols and assessed using different outcome measures (Saltzman et al., 2020). More importantly, it suggests the core clinical question is not whether a given regenerative therapy is universally successful, but rather which specific patients will respond to a particular product under defined biological or biomechanical conditions.

A major contributor to this uncertainty is the dual heterogeneity of both the patient and the therapeutic product. Patient-level variability encompasses disease stage, inflammatory phenotype, metabolic status, mechanical loading, structural damage patterns, comorbidities, and molecular endotypes (Arbeeva et al., 2023; Tang et al., 2025). Product-level variability is equally complex. PRP preparations differ in platelet concentration, leukocyte content, and activation methods. Similarly, cellular products such as MSCs, BMAC, and MFAT vary significantly in tissue source, cell dose, viability, and secretome profiles. Furthermore, EV-based therapies require rigorous characterization of particle number, cargo, surface markers, and isolation methods to improve reproducibility, comparability, and biological interpretation (Welsh et al., 2024). Population-level evidence and conventional eligibility criteria often fail to capture this high-dimensional variability, thereby perpetuating empirical, trial-and-error clinical practices.

Artificial intelligence (AI) and big data analytics offer a methodological framework to transition KOA regenerative medicine from empirical protocols to precision care. By integrating multimodal data spanning clinical variables, quantitative imaging, biomechanics, multi-omics, and product attributes, AI models may help identify mechanism-based endotypes and estimate individual response probabilities (Arbeeva et al., 2023). Currently, AI applications in KOA are largely focused on automated imaging assessment and disease progression prediction. Direct responder-prediction models for orthobiologic therapies remain scarce and generally require independent external validation and prospective clinical evaluation (Oettl et al., 2025).

**FIGURE 1:**
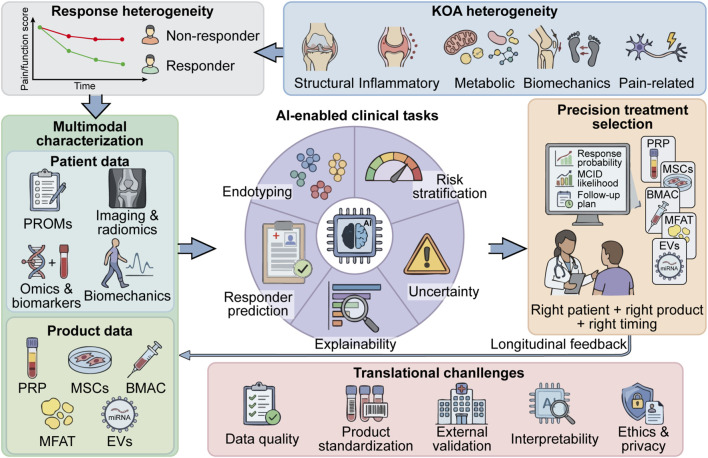
Conceptual framework for AI-guided precision regenerative medicine in knee osteoarthritis. KOA heterogeneity and variable treatment responses arise from patient- and product-level variability. Multimodal characterization of patient data and orthobiologic product attributes can support AI-enabled endotyping, responder prediction, and precision treatment selection, while clinical translation remains limited by data quality, product standardization, validation, interpretability, and ethical governance.

This Mini Review explores AI-guided precision regenerative medicine for KOA. We first discuss how patient and product heterogeneity contribute to inconsistent treatment responses. Next, we examine multimodal characterization as the foundational data source for endotyping and responder prediction. Finally, we evaluate key translational barriers, including model interpretability, workflow integration, and reporting standards such as TRIPOD + AI (Collins et al., 2024). We also outline future directions toward dynamic prediction models, clinical decision support systems, and patient-specific digital twins. The overall conceptual framework of patient–product–response matching is summarized in Figure 1.

## 2 Response heterogeneity in KOA regenerative medicine

Clinical trial evidence clearly illustrates response heterogeneity in KOA regenerative medicine. Earlier systematic reviews and meta-analyses support the potential of MSC-based approaches for pain, function, and selected cartilage-related outcomes, but also emphasize substantial heterogeneity in cell source, preparation, comparators, and outcome assessment (Gong et al., 2021; Maheshwer et al., 2021). However, rigorously designed trials have produced inconsistent or non-superior results compared with established comparators. For instance, the phase 3 MILES trial found that no orthobiologic intervention was superior to corticosteroid injection at 1 year (Mautner et al., 2023). Similarly, the ADIPOA2 phase 2b randomized trial did not establish clear superiority of intra-articular adipose-derived mesenchymal stromal cells over placebo for pain and function (Pers et al., 2025). A randomized comparison between MFAT and PRP showed clinically meaningful improvements in both groups but no clear superiority of one biologic product over the other (Baria et al., 2022). These findings suggest that average treatment effects in heterogeneous trial populations may obscure distinct responder and non-responder subgroups, rather than disproving the biological rationale of regenerative medicine.

Patient-level variability helps explain these inconsistent outcomes. Baseline demographic and clinical factors, including age, sex, disease stage, pain sensitization, inflammatory phenotype, metabolic status, and mechanical loading, may influence therapeutic response. Clinical evidence generally suggests that early-to-moderate KOA without severe malalignment may be more amenable to biologic modulation than advanced disease characterized by extensive structural derangement or severe joint damage. Emerging subgroup analyses also suggest potential demographic modifiers. For example, male patients, particularly those younger than 60 years, may exhibit more favorable responses to autologous cell injections than to corticosteroids (Mautner et al., 2025). However, such findings remain hypothesis-generating. They underscore the need for responder-based analyses but do not yet provide validated multidimensional selection criteria for clinical practice.

Product-level and methodological heterogeneity introduce further complexity and often remain inadequately controlled in trial designs. Orthobiologics are not monolithic entities. PRP preparations vary substantially in platelet dose, leukocyte content, activation strategy, and growth-factor profiles. Mechanistic clinical studies suggest that PRP may alter the synovial inflammatory milieu, matrix-degradation markers, angiogenic growth factors, and selected serum biomarkers, but these changes remain exploratory and have not yet produced standardized responder definitions (Lacko et al., 2021; Tucker et al., 2021). The clinical significance of leukocyte content also remains inconsistent across studies (Park et al., 2021; Di Martino et al., 2022; Berrigan et al., 2025). Similarly, cell-based therapies differ substantially in source tissue, cellular composition, viability, senescence, and secretome dynamics (Sun et al., 2024). These biological variations are further amplified by disparate eligibility criteria, injection protocols, and outcome metrics (Saltzman et al., 2020). Without precise product characterization, defining an equivalent “dose” or “intervention” remains elusive.

Taken together, response heterogeneity arises from the complex interaction of patient phenotypes, product attributes, and study designs. Traditional population-level analyses are poorly suited to untangle this high-dimensional variability, providing a clear rationale for AI-guided precision regenerative medicine.

## 3 Multimodal patient and product characterization

Overcoming response heterogeneity requires moving beyond empirical clinical grading toward high-resolution multimodal characterization. On the patient side, deep phenotyping integrates molecular profiling, quantitative imaging, and biomechanical data to capture disease activity often invisible on conventional radiographs. Synovial multi-omics identify alterations in energy metabolism, collagen turnover, and osteogenic proteins as candidate signatures of KOA activity (Ge et al., 2025). Systemically, integrating gut metagenomics with serum and plasma omics links gut–joint axis dysregulation to MRI-detected synovitis (Wang et al., 2026). Spatial omics further classify synovitis into distinct immune–metabolic trajectories, indicating that inflammation requires subclassification beyond simple presence or absence (Zhu et al., 2025).

Quantitative imaging and biomechanics provide complementary structural and functional dimensions. MRI-based radiomics extracts sub-visual texture features to quantify cartilage integrity, subchondral bone remodeling, and early matrix-level changes. In particular, cartilage T2 mapping-based radiomics provides a quantitative route to characterize biochemical cartilage degeneration before gross morphologic loss becomes apparent (Xue et al., 2022; Davey et al., 2024; Gao et al., 2024; Fu et al., 2025). AI-based 3D MRI analysis has further linked AI-determined KL grade with medial meniscus extrusion and regional cartilage thickness, showing how automated imaging can quantify structural heterogeneity beyond conventional radiographic grading (Sekiya et al., 2023). Furthermore, biomechanical measures such as gait kinematics, joint loading, and wearable-derived activity data may explain why patients with similar molecular phenotypes respond differently to localized interventions.

Product-side profiling should convert orthobiologics from loosely defined biological suspensions into quantifiable therapeutic inputs, but these attributes should not be treated as equally validated predictors of clinical response. For PRP, platelet concentration and total platelet dose currently have the strongest preliminary outcome-linked support, with recent meta-analyses suggesting that higher platelet doses may be associated with more durable pain relief and functional improvement in KOA (Bensa et al., 2025; Berrigan et al., 2025). For cell-based therapies, cell dose is also a critical product attribute, although its relationship with clinical outcomes appears non-linear and product-dependent. BMAC dose-escalation data suggest that cellular dosage may influence radiological outcomes, whereas recent MSC dose-focused analyses indicate that dose optimization, rather than simple dose escalation, may be more clinically relevant (Muthu et al., 2024; Rahmadian et al., 2025). Beyond cell counts, functional potency attributes such as viability, stemness, and senescence burden may help explain cell-product heterogeneity and have shown exploratory correlations with autologous treatment outcomes (Sun et al., 2024).

Other product parameters remain clinically relevant but lack consistent outcome associations. For instance, PRP leukocyte content should be recorded because of its potential influence on local inflammatory activity and post-injection tolerability, yet randomized trials have not consistently demonstrated the superiority of either leukocyte-rich or leukocyte-poor formulations (Di Martino et al., 2022; Romandini et al., 2024). Growth factor and broader secretome profiles may provide additional biological and exploratory clinical information, but their predictive value remains less established than platelet dose-related measures (Park et al., 2021; Del Amo et al., 2022). Broader preparation-, donor-, and manufacturing-related variables, including activation strategies, fibrin architecture, donor immune profiles, tissue source, culture conditions, and manufacturing protocols, remain largely mechanistic or standardization attributes rather than validated clinical predictors (Niemann et al., 2023; Grieco et al., 2025; Ragni et al., 2025). For EV-based products, particle number, size distribution, purity, cargo composition including miRNAs, surface markers, isolation methods, storage conditions, and potency assays are central for quality control and regulatory translation; however, their direct associations with clinical response in KOA remain largely unvalidated (Welsh et al., 2024; Figueroa-Valdes et al., 2025). Consequently, future AI-driven therapeutic matching should prioritize evidence-weighted product attributes, integrating clinically supported, unresolved, and mechanistically plausible variables into matched patient–product–outcome datasets rather than treating all product descriptors as equivalent inputs.

## 4 AI-enabled endotyping and responder prediction

AI-enabled precision regenerative medicine must be organized around specific clinical tasks rather than algorithm categories. In KOA, the most relevant tasks for informing biological treatment selection are endotype discovery, imaging-based risk stratification, and direct treatment-response prediction.

### 4.1 Endotyping: from heterogeneous KOA to treatable subgroups

AI-enabled endotyping aims to identify biologically, structurally, or mechanically coherent KOA subgroups that may differ in progression risk and therapeutic responsiveness. Data-driven biclustering of Osteoarthritis Initiative data has revealed potential KOA phenotypes with distinct symptom, functional, and structural trajectories (Nelson et al., 2022). Molecular phenotyping studies further support this concept, showing that metabolic and inflammatory profiles can define clinically relevant subgroups and that cytokine–outcome associations may be more informative when analyzed within phenotype-defined groups (Calvet et al., 2023; Calvet et al., 2024). More recently, multimodal deep learning using plasma, synovial fluid, and urine multi-omics identified three distinct KOA endotypes and improved classification of post-arthroplasty pain and function (Rockel et al., 2025). Although these models do not yet directly assign regenerative therapies, they provide a useful methodological template for identifying treatable endotypes that could later be linked to orthobiologic response.

### 4.2 Radiomics and imaging-derived risk stratification

Regenerative therapies are unlikely to act uniformly across patients with divergent structural burdens. For example, DeepKOA used multimodal knee MRI from the Osteoarthritis Initiative to predict KOA progression over 24–48 months, illustrating how high-dimensional structural information may support progression enrichment before irreversible joint damage occurs (Hu et al., 2023). Interpretable automated machine learning models have also been developed to predict rapid KOA progression, with particular emphasis on young patients and early-stage disease (Castagno et al., 2025). Recent systematic reviews affirm the promise of these progression models but note substantial heterogeneity in outcome definitions, dataset sources, and validation strategies (Liu et al., 2025). Although these studies provide indirect evidence for regenerative medicine, progression-enrichment models are clinically relevant because they may help identify patients who are more suitable for early intra-articular biologic intervention before structural deterioration becomes irreversible.

### 4.3 Responder prediction: direct evidence remains scarce

The most direct evidence for AI-guided regenerative therapy currently comes from PRP responder prediction. Recent models by Oettl et al. and Zhang et al. both showed promising ability to predict 6-month clinical responses after PRP treatment, but they differed substantially in feature selection and modeling strategy. Oettl et al. developed a PROM-centered model based mainly on baseline clinical and patient-reported variables and used an inherently interpretable explainable boosting machine (EBM). The EBM achieved an AUC of 0.81, with PROMIS Mental, PROMIS Physical, and KOOS JR scores emerging as influential predictors of clinically meaningful improvement (Oettl et al., 2025). In contrast, Zhang et al. constructed a biomarker-enriched model incorporating baseline clinical and blood-derived features, including anthropometric, metabolic, lipid, hepatic, renal, and biochemical indices. They selected a gradient boosting classifier, a less inherently interpretable ensemble method, and applied SHAP analysis for *post hoc* interpretation, identifying osmotic pressure, lipoprotein(a), and uric acid as major contributors to 6-month PRP response (Zhang et al., 2026). These studies suggest that both patient-reported baseline phenotypes and systemic biochemical markers may carry predictive weight. However, their clinical translation remains constrained by single-center retrospective designs, reliance on internal validation, modest sample sizes, and the absence of product-level characterization (Oettl et al., 2025; Zhang et al., 2026).

AI responder-prediction evidence for MSC-, BMAC-, MFAT-, and EV-based therapies remains largely absent. Existing clinical studies provide subgroup signals, dose-response observations, or product-quality associations but do not yet constitute validated AI models for individualized treatment allocation. This distinction is important because a clinically actionable prediction model should estimate treatment-specific response probabilities while integrating patient phenotypes, product attributes, treatment protocols, and longitudinal outcomes. Until matched patient–product–outcome datasets become available, evidence for AI-guided treatment selection beyond PRP should be regarded as hypothesis-generating.

## 5 Clinical decision support and translational bottlenecks

The evidence summarized in Table 1 indicates that model development has outpaced clinical implementation. For KOA regenerative medicine, an accurate prediction model is only an intermediate step. Its clinical value depends on whether it can be translated into a usable clinical decision support system (CDSS).

**TABLE 1:** Evidence map of AI-relevant studies for KOA stratification and regenerative treatment selection.

Study	Evidence category	Data	Therapy relevance	Product-level data	Validation	Limitation
Zhang et al. (2026)	Direct PRP responder prediction; gradient boosting classifier with SHAP interpretation	Baseline clinical and blood-derived features, including anthropometric, metabolic, lipid, hepatic, renal, and biochemical indices; 6-month NRS pain responder	PRP response prediction	No; PRP composition and dose data not included	Internal cross-validation	Single-center; lacks PRP product-level characterization
Oettl et al. (2025)	Direct PRP responder prediction; explainable boosting machine	Baseline clinical and PROM-centered features, including PROMIS, KOOS JR, pain scores, and radiographic grade; MCID achievement	PRP response prediction	No; PRP composition and dose data not included	Internal cross-validation	Retrospective; relies mainly on patient-reported baseline features; lacks PRP product-level characterization
Rockel et al. (2025)	Molecular endotyping	Biofluid multi-omics; post-TKA WOMAC response	Precision stratification	N/A; no orthobiologic treatment exposure was modeled	Internal validation	Predicts arthroplasty response, not biologic therapy response
Castagno et al. (2025)	Progression prediction	Clinical, MRI, biochemical data; rapid progression	Early intervention targeting	N/A; disease-progression model	External validation	Based mainly on public cohorts; not treatment-specific
Nelson et al. (2022)	Data-driven phenotyping	OAI clinical and radiographic data; 96-month trajectories	Trial subgrouping	N/A; phenotyping model without treatment exposure	Exploratory internal analysis	Lacks treatment-response modeling
Hu et al. (2023)	Imaging-derived risk stratification	Multimodal knee MRI; 24–48-month KOA progression	Trial enrichment	N/A; disease-progression model	Internal cross-validation	Predicts natural progression rather than treatment response
Collins et al. (2024)	Reporting framework	TRIPOD + AI	All prediction models	N/A; reporting framework	Guideline	General AI reporting framework, not KOA-specific
Moons et al. (2025)	Bias and applicability assessment	PROBAST + AI	All prediction models	N/A; assessment framework	Guideline	Requires methodological expertise to apply accurately
Vasey et al. (2022)	Early clinical evaluation framework	DECIDE-AI; human factors, safety, utility	Clinical AI-CDSS	N/A; clinical AI evaluation framework	Guideline	Focuses on early deployment rather than long-term efficacy

**Abbreviations:** AI, artificial intelligence; CDSS, clinical decision support system; DECIDE-AI, developmental and exploratory clinical investigations of decision support systems driven by artificial intelligence; KOA, knee osteoarthritis; KOOS JR, knee injury and osteoarthritis outcome score for joint replacement; MCID, minimal clinically important difference; MRI, magnetic resonance imaging; N/A, not applicable; NRS, numeric rating scale; OAI, osteoarthritis initiative; PRP, platelet-rich plasma; PROM, patient-reported outcome measure; PROMIS, Patient-Reported Outcomes Measurement Information System; PROBAST + AI, Prediction Model Risk Of Bias Assessment Tool + Artificial Intelligence; SHAP, shapley additive explanations; TKA, total knee arthroplasty; TRIPOD + AI, Transparent Reporting of a Multivariable Prediction Model for Individual Prognosis or Diagnosis + Artificial Intelligence; WOMAC, Western Ontario and McMaster Universities Osteoarthritis Index.

### 5.1 CDSS design and clinically actionable outputs

A CDSS should not provide simple binary instructions. Useful outputs include treatment-specific response probabilities, the likelihood of achieving the minimal clinically important difference (MCID), uncertainty estimates, contraindications, alternative options, and follow-up recommendations. In musculoskeletal AI, uncertainty quantification has been proposed as a way to flag low-confidence or out-of-distribution predictions, which is essential before applying model outputs to treatment decisions (Vahdani et al., 2025).

### 5.2 Explainable AI and clinician trust

Black-box algorithms are difficult to use for treatment allocation. Explainable AI methods can help clinicians understand why a model predicts benefit or non-response. Common approaches include Shapley additive explanations (SHAP), local interpretable model-agnostic explanations (LIME), feature importance, and explainable boosting machines. In KOA regenerative therapy, explanations should clarify whether predictions are associated with baseline pain, systemic biomarkers, synovitis, structural burden, or product-related features. Existing PRP response models illustrate this principle: Oettl et al. used an explainable boosting machine to predict clinically meaningful improvement after PRP injection, whereas Zhang et al. used SHAP analysis to identify baseline blood and clinical variables associated with PRP response (Oettl et al., 2025; Zhang et al., 2026). However, interpretability does not prove causality. Model explanations should remain biologically plausible and prospectively testable before being used for treatment allocation (Zsidai et al., 2023).

### 5.3 External validation and prospective testing

Validation is the main barrier between prediction and clinical use. Many KOA and orthopedic AI models are based on retrospective cohorts, single-center data, or internal cross-validation, which increases the risk of overfitting and limited generalizability. In regenerative medicine, performance may also vary with imaging protocols, rehabilitation pathways, injection techniques, and product preparation methods. External validation should therefore include geographic, temporal, and multicenter testing, together with calibration, decision-curve analysis, subgroup performance, and missing-data assessment. Reporting should follow TRIPOD + AI, and risk of bias and applicability should be assessed using PROBAST + AI (Collins et al., 2024; Moons et al., 2025). For early live clinical evaluation, DECIDE-AI provides guidance on human–AI interaction, workflow, safety, and clinical utility (Vasey et al., 2022). Randomized evaluations should align with SPIRIT-AI and CONSORT-AI extensions (Cruz Rivera et al., 2020; Liu et al., 2020).

### 5.4 Regulatory oversight and workflow integration

Even validated models may fail if they cannot fit clinical practice. A KOA regenerative CDSS would require governance for data privacy, accountability, model updating, performance monitoring, and local recalibration. Orthobiologic therapies add another difficulty because PRP, MSC, BMAC, MFAT, and EV preparations differ across centers, devices, donors, processing protocols, and release criteria. A model trained on one product formulation may not generalize to another. Integration with electronic health records, imaging systems, laboratory data, product-quality reporting, and longitudinal outcome tracking is therefore essential. Recent orthopedic and musculoskeletal AI reviews emphasize that deployment requires workflow compatibility, monitoring for algorithm drift, adaptive governance, and prospective evaluation rather than accuracy metrics alone (Loftus et al., 2023; Glinkowski et al., 2026).

The implementation threshold is therefore not a higher AUC alone. A useful KOA regenerative CDSS must show that its predictions are explainable, calibrated, generalizable, safe in live settings, and linked to standardized product characterization.

## 6 Future directions

Future progress in AI-guided regenerative medicine for KOA will depend less on building increasingly complex algorithms than on creating datasets that connect clinically meaningful variables. The immediate priority is to establish matched patient–product–outcome cohorts that capture clinical phenotype, PROMs, radiomics, biomechanics, molecular biomarkers, orthobiologic product attributes, treatment protocols, adverse events, and longitudinal outcomes. Such datasets would allow future models to test whether specific inflammatory, metabolic, structural, or biomechanical endotypes respond preferentially to defined PRP, MSC, BMAC, MFAT, or EV formulations. An analogous template is provided by the IMI-APPROACH study, which used machine-learning-assisted recruitment to enrich OA trials with patients more likely to experience structural or symptomatic progression (Widera et al., 2023). Precision orthobiologics frameworks have emphasized the need to move from diagnosis-based intervention toward biomarker-informed regenerative strategies, but prospective therapy-matching datasets remain limited (Navani et al., 2025).

A second direction is dynamic prediction. Most current models estimate response before treatment, whereas KOA evolves over time across pain, function, structure, and inflammation. Future systems should update response probabilities using serial PROMs, quantitative MRI, biomarkers, wearable-derived gait or activity data, and early post-treatment response. This approach could support decisions such as whether to repeat PRP, switch to another orthobiologic product, intensify rehabilitation, or discontinue ineffective biological treatment. AI-enabled KOA decision aids using PROMs and clinical data have already shown the feasibility of personalized decision support, while longitudinal modeling frameworks from related rheumatic diseases provide templates for tracking response trajectories (Falck et al., 2024; Jayakumar et al., 2025).

Digital twins and virtual trials represent a longer-term extension of this framework. Quantitative MRI-based digital twin models are beginning to link deep-learning segmentation, imaging biomarkers, OA incidence, and knee replacement risk, providing a structural basis for individualized disease simulation (Hoyer et al., 2025). In regenerative medicine, such systems could eventually simulate expected trajectories under different product types, doses, and retreatment schedules. However, this remains aspirational until models incorporate real-world orthobiologic exposure and undergo multicenter validation.

Reinforcement learning may further support adaptive treatment strategies by learning sequential decisions from longitudinal data. In principle, it could help optimize combinations of biologics, rehabilitation, analgesia, and monitoring intervals. However, current clinical reinforcement learning evidence is concentrated outside KOA and regenerative musculoskeletal care, and major barriers remain in reward definition, safety, interpretability, and data requirements (Frommeyer et al., 2025). Therefore, reinforcement learning should be framed as a future research direction rather than a near-term clinical tool. The broader aim is to move from static responder prediction toward adaptive, explainable, and prospectively validated precision regenerative care.
